# The En-Gedi Spring Site and the Judahite Expansion into the Judaean Desert in the Late Iron Age

**DOI:** 10.1080/03344355.2023.2190273

**Published:** 2023-06-09

**Authors:** Avraham Mashiach, Uri Davidovich

**Keywords:** Judahite monarchy, Iron Age IIB–IIC, En-Gedi, Dead Sea, Judaean Desert, Neo-Assyrian period

## Abstract

This article discusses the results of the excavations conducted in the Iron II site near the En-Gedi Spring in 1961–1962 and 2019. The site, consisting of a prominent stone platform documented as early as the 19th century and other recently discovered structural remains, is interpreted as a Judahite outpost built in a strategic location within the oasis of En-Gedi. On the basis of the ceramic assemblage, it is suggested that this site was founded during the early 7th century BCE and was abandoned before the end of that century—making it the earliest Iron Age occupation in the oasis. Combined with historical considerations and a regional analysis, the En-Gedi Spring site enhances our understanding of the Judahite expansion into the Judaean Desert during the late Iron Age.

## Introduction

The 7th century BCE is widely regarded as a transformative era in the history of the Southern Levant in the Iron Age (Koch 2018; Lipschits and Čapek 2019; Faust 2021; Cogan 2021). Sheltered under imperial domination, the now subordinate polities of Judah and the Philistine cities had recovered from the aftermath of the Assyrian campaigns of the late 8th century BCE and witnessed a period of political stability and economic growth, evident in the emergence of specialised regional economies (Faust and Weiss 2011; Finkelstein, Gadot and Langgut 2022). In Judah, this process was accompanied by further elaboration of writing and administrative systems (e.g., Lipschits, Sergi and Koch 2011; Finkelstein 2020) and the establishment of numerous new settlements, farms, forts and outposts in previously sparsely exploited regions (Finkelstein 1994; Stern 2001: 130–215; Thareani-Sussely 2007; Faust 2008). This prosperous episode was, however, short-lived: toward the end of the 7th century BCE Judah and Philistia were thrown into turbulence, which eventually led to their demise.

One region in which the transformations of the 7th century BCE are most evident is the eastern fringe of Judah—the Judaean Desert and the western coast of the Dead Sea. Here, the region that was desolate and barren until the end of the 8th century BCE experienced an unprecedented wave of activity, including the establishment of agricultural estates in the oases of the northwestern Dead Sea shore and the Hyrcania Valley (al-BuqeꜤah) and numerous forts and towers in the desert uplands (Stager 1976; Stern 1994; Ofer 1998; Lipschits 2000; Davidovich 2014: 249–271). Delineating the timing and progress of this revolutionary process is a difficult task, heavily dependent on a chronological assessment of ceramic horizons sourced in excavated, multi-layered, central Judahite sites, as well as on divergent historiographical paradigms for the history of the Judahite monarchy in the last two centuries of its existence (e.g., Na
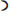
aman 1991; Finkelstein 1994; Stern 2001; Thareani-Sussely 2007; Faust 2008; Lipschits 2019).

The key site for reconstructing the Judahite expansion into the Judaean Desert is, evidently, En-Gedi—the most prominent oasis along the western shore of the Dead Sea (Josh 15:62; Song of Songs 1:14; 2 Chron 20:2). Here, on a low hill in the southern part of the oasis plain (Tel Goren), a thriving Judahite centre was founded during the late Iron Age (Stratum V), on what would become the main mound in the oasis in subsequent centuries. Since the first publications of the Hebrew University excavations at Tel Goren in the early 1960s, it has been suggested that the late Iron Age activity in the oasis of En-Gedi began no earlier than the last third of the 7th century BCE (Mazar, Dothan and Dunayevsky 1966; Stern 2007: 362). While some have challenged this date (e.g., Ussishkin 2011), conventional wisdom still holds that Tel Goren Stratum V is one of the principal sites associated with the post-Assyrian era, eventually destroyed in the Babylonian conquest.

**Fig. 1:**
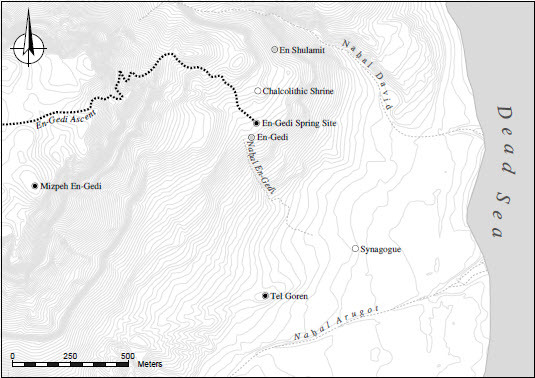
Map of the En-Gedi oasis (Iron Age sites marked in black; major springs and sites from other periods are indicated; note that the ancient route of Nahal En-Gedi in the plain west of the Dead Sea is unknown)

This article reexamines the history of late Iron Age En-Gedi through the lens of the long-neglected site near the En-Gedi Spring (Fig. 1). This site, consisting of a massive stone platform and remains of additional structures, was briefly excavated by two expeditions, separated by more than half a century. On the basis of data from the two excavations, it is argued that this site was the earliest Judahite outpost built in the oasis of En-Gedi and that it thus serves as an important marker for the reconstruction of territorialisation processes in the eastern fringe of Judah during the 7th century BCE.

## The site and its exploration

The En-Gedi Spring site is located less than 50 m northeast of the spring, 195 m below Mediterranean Sea level and ca. 180 m above the oasis plain; it lies ca. 800 m north of Tel Goren as the crow flies (Fig. 1). The site is located on the southern part of a natural terrace, stretching ca. 300 m from Naḥal David in the north to Naḥal En-Gedi, a small gully draining the En-Gedi Spring towards the plain, in the south (ITM 597216/236918). The terrace, one of several created by the stepped western fault of the Dead Sea Transform that moderate the lower part of the Dead Sea Escarpment in the En-Gedi region, is underlain by Cenomanian limestone and dolomite covered by Pleistocene-Holocene conglomerates, tufa and debris flows (Raz 1983). The En-Gedi Spring, one of four large springs irrigating the oasis, provides a constant water supply estimated today at 50 m^3^ per hour. The spring supports dense vegetation of mixed Saharo-Arabian and Sudanian elements, which, before recent pumping, covered a much larger area towards Naḥal En-Gedi and the oasis plain (Danin 2007; Blecher 2012).

Accounts of the remains of a large stone platform in the vicinity of the spring, along with other ancient ruins, were reported as early as the 19th century by travellers who visited En-Gedi (e.g., Robinson 1841: 210; de Saulcy 1854: 180; Tristram 1865: 299; Abel 1911: 136) and particularly by the surveyors of the Survey of Western Palestine, who described the site as the ‘most remarkable ruin’ in the oasis (Conder and Kitchener 1883: 387). Archaeological investigation of the site began in the mid-20th century, within the framework of the first surveys in the oasis conducted by Benjamin Mazar (then Maisler) and Yohanan Aharoni. The latter documented and measured the platform and, based on pottery collected from the surface, suggested a dating in the Iron II, dubbing it ‘the Israelite Tower Near the Spring’ (Aharoni 1958: 29–30; cf. Maisler 1949: 27; see also Naveh 1958: 12, 24; Hadas 2012: site 176). In these surveys additional remains from various periods were documented in the vicinity of the site; some of these were later excavated and studied in detail. They include a Roman–Byzantine lime kiln ca. 30 m northwest of the platform (Hadas 2007), a medieval watermill east of the spring (Hadas 2001–2002), and the famous Chalcolithic ritual complex built on a spur overlooking the spring (Ussishkin 1980).

The first excavations of the Iron Age site near the En-Gedi Spring were conducted in 1961 and 1962 within the framework of the Hebrew University expedition to the oasis expedition). This operation took place at the same time as the excavations at Tel Goren and other sites within the oasis (Mazar, Dothan and Dunayevsky 1966: 13) and was included in Area E, designating the area immediately to the north of the spring. The results of these excavations were never published, except for a concise encyclopaedic description (Mazar 1976: 378). Nevertheless, materials from the Mazar expedition stored in the archives of the Israel Antiquities Authority and the Hebrew University of Jerusalem, including field journals, preliminary plans and photographs, as well as pottery sherds, allow for a detailed reexamination of the excavation results.

In 2019, renewed small-scale excavations were conducted at the site by the authors within the framework of a regional research project (DEADSEA-ECO) headed by Nimrod Marom, a project that explores Holocene human–environment interactions in the Judaean Desert (Lazagabaster *et al*. 2021).^1^ The renewed excavations included several probes dug to the north and west of the platform, beyond the borders of the excavations conducted by the Mazar expedition. The renewed excavations unearthed hitherto unknown architectural remains associated with Iron Age pottery, providing significant new data related to the formation processes, chronology and function of the site.

## Site layout and formation processes

### The stone platform

**Fig. 2:**
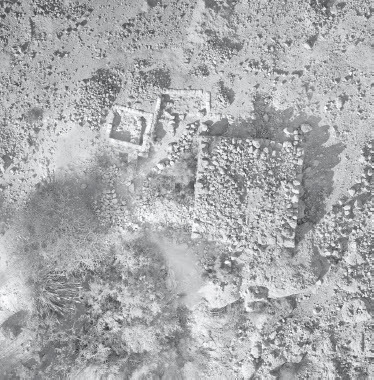
Aerial view of the En-Gedi Spring site during the renewed excavations (photo by Tal Rogovski)

**Fig. 3:**
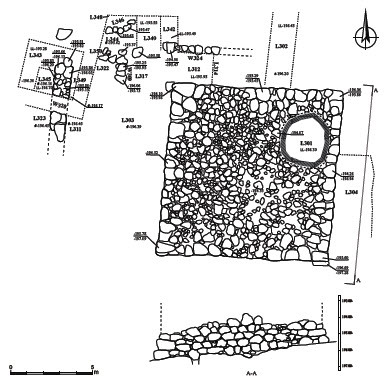
General plan of the site and excavated areas and side view of the eastern wall of the platform

The main architectural feature in the En-Gedi Spring site is a large, almost square, solid stone platform, covering an area of ca. 110 m^2^ (Figs. 2–3). The platform’s four external walls, each 10.5 m long, were built of large, partially dressed limestones (average length of ca.1 m) and were preserved to a height of four to five courses (maximum 2.6 m). Massive stones, ca. 1.5 m long each, were placed in the corners. The stones of the first course protrude slightly outward, creating a stepped foundation (side view A–A in Fig. 3). The inner part of the platform is a dense fill of small to medium-sized fieldstones with little sediment.

**Fig. 4:**
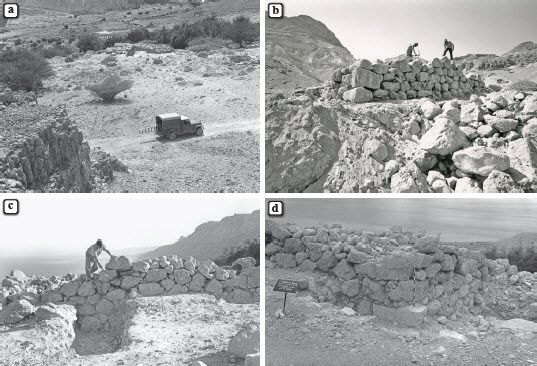
The excavations of the En-Gedi Spring site by the Mazar expedition (1961–1962); a) general view, looking southeast; the site is located near the top of the image, just in front of the vegetation-rich area; b) excavations on top of platform; c) northern wall of platform and L302, looking south; d) northwest corner of platform and L303, looking southeast

Excavations inside the platform, carried out by the Mazar expedition in 1961 and 1962, included the removal of a thin accumulation on top of the platform (L507), followed by excavation of a circular probe (L301), ca. 2 m in diameter, near its northeastern corner (Fig. 4b).^2^ In this probe, the platform’s inner fill was excavated to a depth of ca. 2 m, down to bedrock. The absence of evidence for internal dividing walls or accumulations associated with *in situ* activities inside the platform, along with the density of the stone fill, confirmed the preliminary assumption that the structure consists solely of a raised solid platform.

Further excavations along the outer walls of the platform were conducted by the Mazar expedition in three probes near its northern, western, and eastern walls, labelled L302, L303 and L304, respectively, in which the platform’s outer foundations were exposed. These excavations demonstrated that the platform was erected directly on bedrock, atop a natural rocky outcrop protruding from the terrace surface. According to assessments in the field journal, this outcrop was hewn and flattened by the platform’s builders prior to its construction, but no evidence for this was found in the renewed investigation of the site.^3^

## A northern extension of the platform

**Fig. 5:**
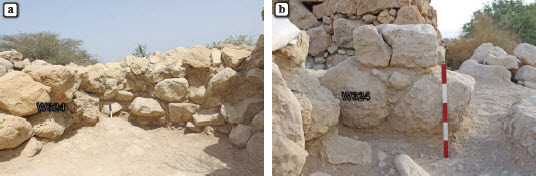
Stone revetment near the northern wall of the platform; a) looking east, with the inner face of Wall 324 visible on the left; b) the outer (northern) face in the western edge of W324, looking south

L302 was a narrow trench excavated by the Mazar expedition perpendicular to the northern outer wall of the platform, to a distance of 3 m. A description provided in the excavation log recounts an exceptional amount of ‘stone collapses’ in this locus and mentions a ‘course of stones’ that runs ‘parallel to the outer wall of the tower’.^4^ Upon our return to the site in 2019, we observed a clear wall line protruding slightly from the surface, parallel to and ca. 2 m north of the northern face of the platform. Renewed excavations conducted near the northwestern corner of the platform, ca. 2 m west of L302, made it clear that this feature is essentially a retaining wall (W324), made of one row of large, partially dressed stones preserved to the height of ca. 0.6 m (two to three courses), that supports a revetment, 2 m wide, abutting the platform’s northern wall. In a clean section made between W324 and the platform (L312), northeast of Mazar’s expedition L303, it was demonstrated that this feature incorporates a dense fill of medium-sized fieldstones and cobbles (L314; probably the ‘stone collapses’ mentioned in the 1960s account), with relatively little sediment and containing few Iron Age sherds (Fig. 5). The western edge of this feature, while slightly damaged following the previous excavations, seems to be in line with the northwestern corner of the platform.

The architectural purpose of this stone revetment cannot be determined with certainty due to the limited exposure and meagre architectural information collected thus far. It should be emphasised, however, that similar constructions do not exist along other faces of the platform and that topographically, the most convenient approach to the platform is from the north. It may thus be suggested that this feature was intended to serve as a base for a ramp or staircase leading up to the platform (cf., e.g., the ramp in the Late Iron Age fort of ꜤEin et-Turaba; Bar-Adon 1989: 42–43), although alternative interpretations (e.g., a secondary expansion of the platform northwards) cannot be ruled out.

## Structural remains northwest of the platform

The 2019 excavation focused on the area to the west and northwest of the platform, following the detection of hitherto unknown ancient architectural remains in this area.^5^ The excavations, extending from the irregular edges of Mazar expedition’s L303 to the west and northwest (covering a total area of ca. 45 m^2^), exposed new architectural remains associated with *in situ* Iron Age deposits and confirmed our initial hypothesis regarding the extension of Iron Age remains in this area. These remains were covered by a thick (Ꜥ 0.5 m) geological layer consisting of compacted silts, sands, gravel and cobbles, interpreted as debris flow, i.e., colluvial deposits induced by heavy torrential rainstorms over the nearby steep western Dead Sea Escarpment (Ben David-Novak, Morin and Enzel 2004). This accumulation ‘sealed’ the ancient remains, and, if not for the 1960s excavations that removed part of this debris and exposed the edges of the features to be described below (without noticing them), it is unlikely that we would have suspected the presence of these remains.

**Fig. 6:**
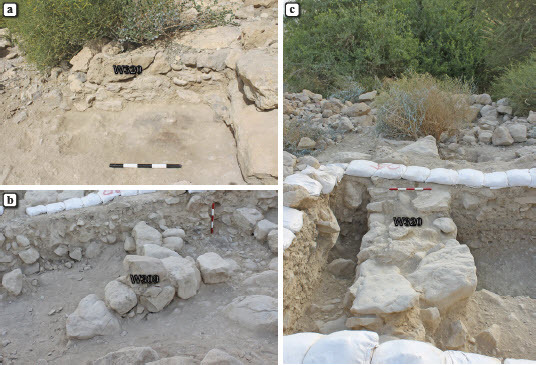
Structural remains northwest of the platform; a) a layer of ashy sediment near the southern edge of W320, looking west; b) W309, looking west (note the thick debris flow in the section west of the wall); c) W320, looking south

The most prominent of the newly exposed remains is W320—a 1 m wide wall built of two rows of medium-sized and large fieldstones and cobbles in a north–south orientation, parallel to and ca. 6.5 m to the west of the western face of the stone platform (Fig. 6). In the renewed excavation, a 5 m long segment of W320 was uncovered, preserved to a height of 0.8 m. On both sides of the lower course of the wall, in limited probes excavated below the debris flow, remains of activity surfaces were unearthed, consisting of thin (ca. 5 cm) sub-horizontal layers of dark grey (ashy) sediments with numerous Iron Age sherds (L311, L323, L343 and L349; Fig. 6a). These layers appear to rest immediately upon pre-settlement geological deposits.

A small segment of an additional north–south wall, W309, was exposed ca. 3 m east of W320 and at a similar distance west of the platform. This wall was built as a single line of medium-sized fieldstones and was poorly preserved to a maximal height of 0.5 m and a length of 2 m (Fig. 6b); its southern edge may have been damaged during the excavation of L303 by the Mazar expedition. As in the case of W320, thin layers of grey ashy sediments containing pottery sherds were found near the base of W309, on both sides (L317, L322 and L344); similar layers had already been observed in L303 according to notes made by the Mazar expedition. North of W309, a semi-circular stone feature was exposed (L346). It was initially interpreted as an artificial construction, but further excavation suggested that it was part of the debris flow and was not associated with cultural deposits.

The limited excavations conducted in 2019 do not permit a comprehensive reconstruction of the nature of the Iron Age activity in the En-Gedi Spring site. Except for limited ceramic assemblages, no other artefacts were retrieved, and the preservation of organic materials (e.g., animal bones) was limited to tiny unidentified fragments, possibly due to moist and saline conditions in the sub-surface associated with the nearby spring. Nevertheless, the exposure of hitherto unknown architectural remains associated with *in situ* deposits dated to the Iron Age clearly suggests that the square platform was not an isolated feature, as previously assumed. As for the relations between the platform and the newly discovered architecture, the removal of the accumulation west of the platform by the Mazar expedition (L303) and the insufficient documentation related to this operation make it impossible to establish a stratigraphic connection between them. Nonetheless, the lack of evidence for chrono-stratigraphic phasing at the site coupled with the homogeneous character of the pottery assemblage found by both expeditions (see below) suggest that all the architectural elements at the site belong to a single, probably short, phase of activity and possibly to a single architectural complex.

## The ceramic assemblage

**Fig. 7:**
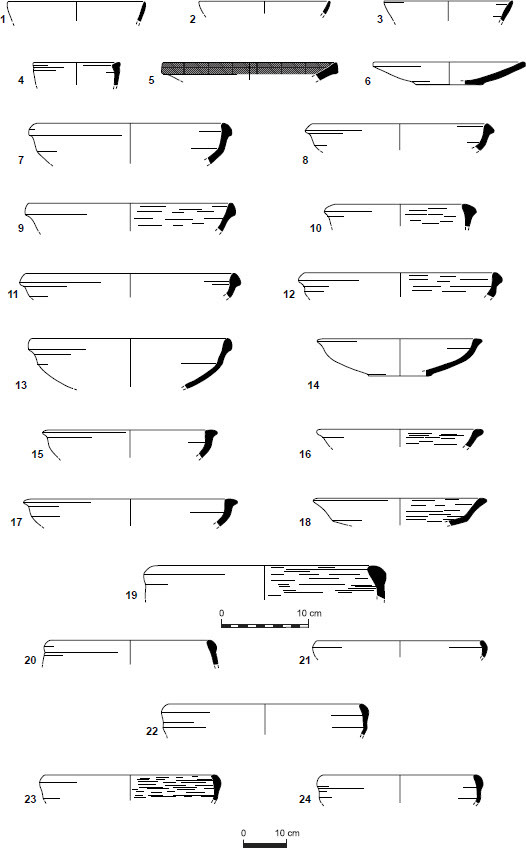
Selected pottery from the site: open forms

**Fig. 8:**
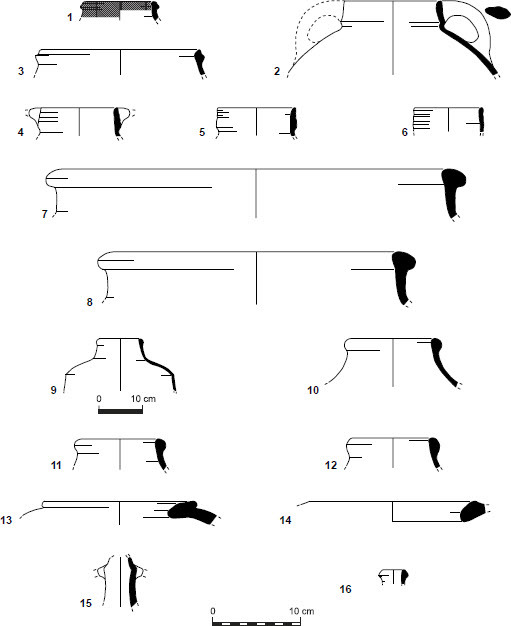
Selected pottery from the site: closed forms

The ceramic assemblages from the En-Gedi Spring site collected by the two expeditions (Figs. 7–8) contain various vessel forms that correspond to the well-known Iron IIB–C ceramic horizons in Judah and its environs (see Aharoni and Aharoni 1976; Zimhoni 1990; Herzog and Singer-Avitz 2015; Gitin 2015).^6^ Some of the forms, such as straight-walled bowls (Fig. 7:1–3), shallow bowls (Fig. 7:5–6), and large bowls (or kraters, Fig. 7:19–24), have a relatively long timespan and are prevalent throughout the Iron IIB–C. In tandem, two groups of forms carry specific chronological significance. The first includes vessels such as the small closed cooking pot with upright neck and moulded ridged rim (Fig. 8:1), cooking pots with upright multi-ridged neck (Fig. 8:4–6) and bowls with outfolded triangular rim (Fig. 7:7–12). These forms are generally considered to be more characteristic of the late Iron IIB repertoire, commonly referred to as the ‘Lachish Level III horizon’ of the late 8th century BCE. The second and perhaps more important group includes pottery types described by several scholars as transitional—serving as a bridge between the forms of the Lachish Level III horizon and those of the late 7th/early 6th century BCE (the ‘Lachish Level II horizon’). This group includes the open cooking pot with a slightly everted ridged rim (Fig. 8:3; see De Groot and Bernick-Greenberg 2012: 68; Freud 2018: 142–143), the closed cooking pot with a prominent single ridge at the bottom of its neck (Fig. 8:2; see Freud 2018: 141), and perhaps some of the cooking pots with a multi-ridged neck, exhibiting a slightly everted rim and a pronounced lower ridge (Fig. 8:4–5; see De Groot and Bernick-Greenberg 2012: 71; Gitin 2015: 347–348, n. 5). In addition, one should note that forms that constitute hallmarks of the Iron IIC, such as the open neckless cooking pot with grooved rim (known as the ‘En-Gedi’ cooking pot due to its prevalence in the Tel Goren Stratum V assemblage), the small standardised bowl with outfolded rim and the bag-shaped storage jar, are missing from the assemblage. These forms frequently appear at sites belonging to the Lachish Level II horizon, including Tel Goren Stratum V (see Yezerski 2007: Pls. 1, 5, 9:4–6), and their absence from the En-Gedi Spring site is of clear significance.

**Fig 7:** Selected pottery from the site: open forms

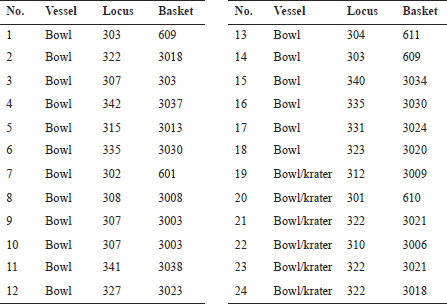

**Table:** 

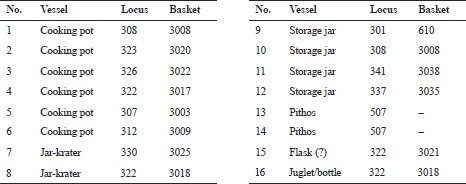

The ceramic typology of the site, coupled with the relatively short duration of activity indicated by the limited anthropogenic accumulation and refuse, seems to suggest that the En-Gedi Spring site was active most probably during the first half of the 7th century BCE. The assemblage is comparable to other Judahite sites with strata from this period, particularly in Jerusalem (City of David Strata 12–11: De-Groot and Bernick-Greenberg 2012; cf. Phase II in Dan-Goor 2022) and its environs (e.g., Gadot *et al.* 2019; Billig, Freud and Bocher 2022; Sapir *et al.* 2022; cf. also ꜤAroer Stratum IIa: Thareani 2014). Indeed, most forms in the assemblage can also be found in late 8th-century BCE sites, but can be equally associated with the early 7th century BCE. As has been compellingly stressed by many scholars (e.g., Finkelstein 1994: 169–172; Finkelstein and Na
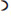
aman 2004; Davidovich 2014: 257–259; Lipschits 2019: 13–18; van der Veen 2020), it is clear that the production and use of Iron IIB forms did not cease immediately after the destructions that mark the Lachish Level III horizon, but instead, continued during the first decades of the 7th century BCE, especially in regions that did not suffer destruction, such as the Judaean Highland. In other words, the Judahite pottery repertoire of the first half of the 7th century BCE is expected to show much resemblance to typical Iron IIB assemblages, concurrently with a gradual introduction of forms that would become typical in the Iron IIC. This picture is in keeping with the assemblage from the En-Gedi Spring site, and therefore suggests, in conjunction with historical considerations (see below), that the site should be dated to the beginning of the 7th century BCE.

The assemblage from the En-Gedi Spring site can be clearly contrasted with that of Stratum V at Tel Goren, which is a typical (late) Iron IIC assemblage. Interestingly, the notion that the site near the En-Gedi Spring is earlier than Tel Goren Stratum V was already implied in the field journals of the Mazar expedition. In the March 14, 1962 entry, in reference to the finds from the Spring site, the excavators state that: ‘The few sherds [from the excavations in the platform] give the impression that they are earlier than the vessels of Stratum V at Tel Goren and ended together with it’.^7^ The same view was expressed in the March 24 account, where it was concluded that: ‘We do not have yet a clear picture of the pottery, yet some sherds suggest that the structure [i.e., the platform] began before the settlement of Tel Goren … ’. For reasons unclear, however, these preliminary observations were later omitted from the brief summaries of the excavations written by Mazar (1976: 378; 1993: 405), in which the platform was presented as contemporary with Tel Goren Stratum V.

## Discussion

**Fig. 9:**
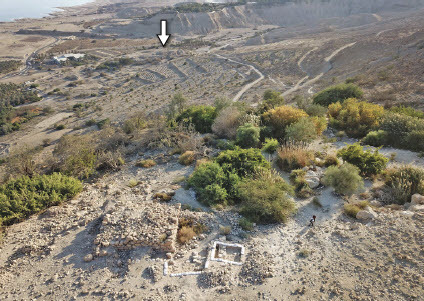
The site near the En-Gedi Spring, looking southeast (photo by Tal Rogovski); Tel Goren is visible in the oasis plain (marked with white arrow)

The data presented in this article suggests that the area of the En-Gedi Spring housed, during the early 7th century BCE, the earliest Judahite occupation in the En-Gedi oasis. The location of the site must have been carefully selected, as it combines three important qualities: 1) proximity to the main spring irrigating the central part of the oasis; 2) a commanding view of the lion’s share of the oasis, especially to the south and southeast towards the outlet of Naḥal ꜤArugot (the area surrounding Tel Goren; Fig. 9); and 3) the starting point of the major overland route connecting the oasis to heartland Judah. This route, the eastern segment of which is now known as the En-Gedi Ascent (Arabic: Naqb ꜤAyn Jidi), rises from the spring to the top of the Dead Sea Escarpment through a steep winding path (Fig. 1) and connects the oasis, through a system of desert trails, to multiple destinations in the Judaean Highland (Harel 1967: 22–24; Feldman 1973; Ilan 1973; Amit 1992; cf. the late Iron Age fortlet on top of the En-Gedi Escarpment [Shaqrat an-Najjar]: Meshel and Ofer 2008). This unique combination of constant availability of water, an effective viewpoint and high connectivity made the En-Gedi Spring the terrestrial gateway of the oasis. The construction of a site with a massive stone structure at this location must therefore be interpreted as a calculated strategic act, intended to gain (and maintain) domination over the oasis.

The remains of the Iron Age site near the En-Gedi Spring, with specific emphasis on the large, skillfully built platform, suggest that the site was founded as a royal Judahite initiative, rather than as an act of ‘civic’ settlement expansion. While the extent of the site to the north and northwest is as yet unknown, it seems to have been rather small (ca. 0.1–0.2 hectares?) and apparently comprised a single architectural complex. The small size of the site suggests that it did not house a large sedentary community and probably served other functions. This is also indicated by the very thin anthropogenic accumulations and the strikingly restricted quantity and diversity of refuse deposited at the site, which may testify to a short occupation, possibly lasting no longer than several decades.

The solid raised platform, which drew the attention of travellers and archaeologists since the early days of exploration at En-Gedi, is the most notable feature at the site. Its construction method, involving the use of a wide dense stone mass supported by strong single-faced walls, is widely attested in public constructions in Judah and the Southern Levant in the Iron II. It was employed primarily in urbanized contexts, featuring prominently in fortification walls and towers (e.g., in Jerusalem: Avigad and Geva 2000; Tell en-Naṣbeh: McCown 1947: 191) and in elevated royal administrative buildings (Sharon and Zarzecki-Peleg 2006). Isolated raised stone platforms, however, are relatively rare in the Iron II in extramural contexts. An early feature, almost identical to the En-Gedi Spring platform, was uncovered at the Iron I site of Giloh (Mazar 1990: 77–84) and was similarly interpreted as the solid foundation of a tower. Stone platforms are also attested at several sites in the Dead Sea region, including Rujm al-Baḥr (Bar-Adon 1989: 3–14), Rujm esh-Shajara (Bar-Adon 1989: 86; cf. Bar-Adon 1972: 130), and Meṣad Gozal (Aharoni 1964; 1965; Talis 2013). In all three cases, however, the suggested Iron Age date for the existing architectural remains is not supported by sufficient evidence; thus, their chrono-cultural relation to the En-Gedi Spring site is uncertain (Davidovich 2014: 160–162, 170–172, n. 189).^8^

Given the notable size of the platform, the large stones used for its construction and its overall resemblance to fortification architecture, the En-Gedi Spring structure was interpreted by its first explorers as a solid foundation of a tower, the superstructure of which was not preserved (Aharoni 1958: 29–30; Mazar, Dothan and Dunayevsky 1966: 13; Mazar 1976: 378). This notion, although plausible, is not without difficulties. The main problem is that the raised platform does not carry any remains of a stone superstructure, nor was it found surrounded by heavy collapse, as would be expected from an elevated tower. It could be argued that the stones of the superstructure were later robbed for agricultural purposes, e.g., for burning lime in the nearby Roman-Byzantine kiln, but such an interpretation would struggle to explain why the superstructure would have been dismantled in its entirety, while the platform (which contains an immense number of stones) would have been left intact. The possibility that the superstructure was built out of wood (as was suggested, for example, in the case of Giloh; Mazar 1990: 77–84) should also be rejected, as a wooden construction would not have required such a massive stone foundation. Another possibility is that the superstructure was built out of mudbricks that had since collapsed and eroded; indeed, mudbrick constructions on top of massive stone foundations are common in fortification architecture of Iron II sites (e.g., at Lachish: Tufnell 1953: 94–95). Nonetheless, post-depositional processes associated with brick constructions typically result in secondary deposition of the brick debris (compare the neighbouring Chalcolithic shrine: Ussishkin 1980: 14–16). Given that the platform was almost entirely exposed prior to the excavations and that there was a complete absence of brick debris at the site, this possibility remains unfounded.

When considering these difficulties, together with the scarcity of comparable massive stone platforms in contemporaneous Judahite towers and fortlets, including in the Judaean Desert region (cf. Bar-Adon 1972; 1989: 41–48; Mazar 1982; 1990: 96–98; Barkay, Fantalkin and Tal 2002; Meshel and Ofer 2008; Davidovich 2014: 139–160), one may wonder whether the platform served a different purpose that did not incorporate a superstructure. A raised stone platform could have been associated with cultic activities, evoking the biblical concept of *bamah* (Fried 2002: 437–444). If so, it may be hypothesised that construction of a cultic site in this location derived from the same desolate, dramatic scenery and environmental qualities that made it an ideal location for ritual activities millennia before (cf. Ussishkin 1980: 34–38 for the Chalcolithic ritual complex). This interpretation, however, is as yet speculative, given the absence of artefacts and ecofacts that can be associated with rituals. Moreover, arguing that during the early 7th century BCE, when the En-Gedi region was sparsely populated, a Judahite cult site was established here, only to be abandoned a few decades later, raises further historical and socio-cultural difficulties that cannot be resolved by the current evidence.

## En-Gedi and the Judahite expansion to the Judaean Desert

During the 7th century BCE, the Judaean Desert witnessed an unprecedented wave of human activity. Archaeological excavations and surveys indicate that in the course of this century, multiple sites were established in this hitherto barren region. Newly founded sites were investigated along the western coast of the Dead Sea and its oases (En-Gedi: Mazar, Dothan and Dunayevsky 1966; Stern 2007; ꜤEin el-Ghweir: Blake 1966; 1967; Bar-Adon 1989: 33–40; ꜤEin et-Turaba: Bar-Adon 1989: 41–48; Khirbet Mazin/Qasr el-Yahud: Bar-Adon 1989: 18–29; ꜤAin Feshkha-Qumran: De Vaux 1973; Hirschfeld 2004: 66–68; Magen and Peleg 2018; and Rujm el-Bahr: Bar-Adon 1989: 3–14), as well as in the Hyrcania Valley (Cross and Milik 1956; Stager 1976; Master 2009), in the ‘Desert of Benjamin’ east of Jerusalem (e.g., Kh. Shilḥah: Mazar, Amit and Ilan 1996; Vered Yericho: Eitan 1983), and along several desert routes (Bar-Adon 1972; Davidovich 2014: 146–160). Following his excavations at En-Gedi, B. Mazar ascribed the Judahite expansion into the Judaean Desert to the reign of Josiah in the last decades of the 7th century BCE, after the Assyrian withdrawal from the Southern Levant—a notion that was later adopted by other scholars (Mazar, Dothan and Dunayevsky 1966: 16; Stern 1994; 2001: 137; Mazar, Amit and Ilan 1996; Lipschits, Sergi and Koch 2011: 24–26). More recently, following paradigmatic shifts in the historiography of both the Assyrian and post-Assyrian eras (e.g., Na
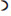
aman 1991; Finkelstein 1994; Faust 2008), several authorities have argued for an earlier, late 8th/early 7th century BCE, date for the beginning of this process, setting the days of Hezekiah or Manasseh as the possible contexts for the Judahite expansion into the desert (e.g., Vaughn 1999: 71–78; Lehmann 2012: 305; and see detailed discussion in Davidovich 2014: 257–263).

Special attention was given in this discussion to the foundation of En-Gedi, i.e., Tel Goren Stratum V, considered to be the main Judahite centre in the Desert of Judah (sensu Josh 15:61–62). Following its excavation in the early 1960s by the Mazar expedition, this stratum was dated to the last third of the 7th century BCE on the basis of historical considerations coupled with the ceramic evidence, which accords well with the date of strata attributed to the Babylonian conquest (Lachish Level II horizon) elsewhere in Judah (Mazar, Dothan and Dunayevsky 1966: 38; Stern 2007: 361–362; Yezerski 2007). This date, however, was challenged by several scholars, who pointed to some artefactual and stratigraphic evidence for the existence of a ‘pre-Stratum V’ phase at Tel Goren as support for a late 8th-century BCE date for the establishment of the site (Ussishkin 2011: 227–229; see also Barkay 1992; 1995). The stratigraphic complexity of Iron Age Tel Goren was indeed manifest in renewed small-scale excavations that we conducted in 2020, but no evidence for the foundation of Stratum V at Tel Goren earlier than the mid-7th century BCE has surfaced to date.^9^

The finds from the En-Gedi Spring site, which provide a clear indication for an earlier Judahite presence in the oasis, shed new light on the foundation process of Iron Age En-Gedi and allow for a more nuanced reconstruction of the expansion of the Kingdom of Judah into the Judaean Desert. The evidence suggests that the En-Gedi Spring site was active during the first half of the 7th century BCE and that its foundation (and possibly also its abandonment) preceded that of Stratum V in Tel Goren. This implies that Judahite control over the oasis of En-Gedi began earlier than previously hypothesised, with the foundation of a relatively small outpost near the spring. This site, with its massive platform erected for either military/administrative or ritual purposes, reflects an incipient Judahite foothold in the major oasis of its eastern wilderness. Its strategic location granted its occupants good control over the oasis and its environmental resources, allowing Judah to establish its presence in this desolate region.

The new data attesting to an early 7th-century BCE date for the beginning of the Judahite occupation of En-Gedi also allows for a better understanding of the enigmatic Hebrew inscription found almost 50 years ago south of Naḥal Yishai, ca. 1.2 km northeast of the En-Gedi Spring (Bar-Adon 1975). This inscription, written in ink in Judaean script on a stalagmite within a small cliff shelter, was dated palaeographically to the late 8th/ early 7th century BCE. While the meaning and context of this inscription remain obscure (*ibid.*; Parker 2003: 270–272), it may now be better contextualised within the broader framework of the Judahite expansion to the oasis and its environs.^10^

The early occupation of En-Gedi, epitomised in the foundation of the En-Gedi Spring site, was evidently one component—albeit a significant one—in the much broader process of Judahite territorialisation under Assyrian hegemony during the early 7th century BCE. A recent reevaluation of all Iron Age sites in the Judaean Desert, which included a detailed inspection of both published and unpublished pottery assemblages and new surveys in multiple sites (Davidovich 2014: 44–79, 139–160, 255–257), demonstrated that the ceramic assemblages in most sites 1) are dominated by Iron IIC forms; 2) contain very few *fossiles directeurs* of the Iron IIB (contra Vaughn 1999: 71–78; Master 2009; Ussishkin 2011: 227–229); and 3) have yielded clear examples of ‘transitional’ Iron IIB–C forms, comparable to those found at the En-Gedi Spring site. These observations have led one of the authors (U.D.) to suggest that the major phase of Judahite expansion to the Judaean Desert occurred during the first half of the 7th century BCE, corroborating earlier assessments that were more reliant upon historiographic notions (e.g., Finkelstein 1994: 175–181; Faust 2008: 170–172). Unlike the En-Gedi Spring site, however, most sites in the Judaean Desert continued in use throughout the 7th–early 6th centuries BCE, as indicated by the dominance of Iron IIC forms in the assemblages.

Toward the mid-7th century BCE, possibly still within the Assyrian era, the centre of gravity in En-Gedi shifted to Tel Goren in the oasis plain. This process may perhaps be attributed to the consolidation of Judahite rule in the Judaean Desert, resulting in a general shift in the nature of the Judahite presence at En-Gedi from isolated outpost to regional economic hub. This reconstruction accords well with the historical processes associated with the period of Assyrian hegemony in the Levant. The initial establishment of En-Gedi in the form of a small outpost near the spring should be attributed to the years that followed the punitive Assyrian campaigns of the late 8th century BCE, during the later part of Hezekiah’s reign or the early days of Manasseh, whose reign is perceived as a prosperous era of demographic and economic growth (e.g., Finkelstein 1994; Knauf 2007). Recent studies have shown that while being a semi-independent Assyrian vassal state, the Kingdom of Judah experienced rapid economic development, reflected in specialised agricultural production (e.g., Eitam 1996; Gitin 1997; Greenberg and Cinamon 2006; Faust and Weiss 2011; Gadot 2015; Sapir-Hen 2017; Finkelstein, Gadot and Langgut 2022), long-distance trade connections (e.g., Finkelstein 1992; Katz 2008: 121–141) and sophisticated administrative systems (e.g., Lipschits, Sergi and Koch 2011; Sapir *et al*. 2022). Against this background, the Judahite expansion to En-Gedi and the Judaean Desert, which began in the early 7th century BCE and was consolidated with the foundation of Stratum V at Tel Goren in the mid-7th century BCE, should be interpreted as part of a broad trajectory aimed at exploring new economic possibilities related to the natural resources of the Dead Sea area, i.e. salt, bitumen and the cultivation of unique cash-crops (date palms and possibly perfume plants; see Mazar, Dothan and Dunayevsky 1966: 20–21; Stern 1994; Davidovich 2014: 263–268). This trajectory not only established En-Gedi as an important economic hub during the last century of Iron Age Judah, but also shaped the history of the oasis and the Dead Sea region for centuries to come.

## Notes

1 The excavations, carried out in April and December 2019, totalled ten days (with 5–10 excavators).

2 The 1961 excavations at the site were recorded using one locus number, L507 (no baskets were used). The 1962 excavations were recorded using locus numbers L301–L305 and basket numbers B601–B612. The 2019 excavations were recorded using locus numbers L307–L350 and basket numbers B3001–B3045.

3 In addition to the excavations attached to the platform walls, the Mazar expedition uncovered a natural rock fissure below the platform. The two edges of this fissure, described in the excavation journal as a ‘rock-hewn channel’, were discerned beneath the western and eastern faces of the platform. In both cases, large stones had to be placed across the fissure’s aperture to ensure stable construction of the outer walls. The western edge of the fissure was excavated by the Mazar expedition to a depth of ca. 3.2 m (L305), exposing a thick accumulation of grey sediments and small stones. This operation initially led the excavators to speculate that this feature was a manmade channel running beneath the platform’s foundations, although its purpose was not discussed in the field journal. Its omission from Dunayevsky’s final plan and from Mazar’s overviews (Mazar 1976; 1993) suggests, however, that this hypothesis was later ruled out. As far as can be assessed from the available data, this feature is indeed a natural fissure not modified or used by the builders of the platform. The lack of artificial modification, coupled with the absence of cultural remains in the fissure, supports this conclusion (see, further, Mashiach 2022).

4 The quotation is from the field journal of the Mazar expedition, stored in the archives of the Israel Antiquities Authority; translated from Hebrew by the authors.

5 Architectural remains that clearly post-date the Iron Age are beyond the scope of this article.

6 The pottery assemblage from the Mazar expedition includes at least seven diagnostic sherds, kept in the Israel Antiquities Authority archives, out of an unknown number of sherds collected during that operation. The assemblage of the renewed excavations includes a total of 61 diagnostic rim sherds.

7 Translated from Hebrew by the authors. The original entry in the excavation log mentions Stratum IV instead of Stratum V, as at the time of writing, Persian period remains had not yet been found at Tel Goren and the Iron Age remains were therefore assigned to Stratum IV. This initial formulation was ‘corrected’ here in alignment with current conventions.

8 In Rujm al-Baḥr and Meṣad Gozal, the raised platforms are clearly related to their proximity to the Dead Sea level and the need to stabilise their foundations (e.g., Oron *et al*. 2015). Both sites also contain clear remnants of stone superstructure, which in one case (Rujm al-Baḥr) is dated to the Late Hellenistic and Early Roman periods (and see Hirschfeld 2006 for Meṣad Gozal). Rujm esh-Shajara, an isolated structure on the Dead Sea shoreline between Khirbet Mazin/Qasr el-Yahud (Meṣad Qidron) and ꜤEin al-Ghweir (ꜤEinot Qaneh), is perhaps the closest parallel (albeit smaller and rectangular in shape, ca. 8.7 × 6.1 m) to the En-Gedi Spring structure, although it was not understood as such by its excavator (Bar-Adon 1989: 86) and its date remains enigmatic due to the dearth of material remains at this site (Davidovich 2014: 170–172).

9 A detailed reevaluation of the chrono-stratigraphy of Tel Goren in its early phases is beyond the scope of the present paper. Nonetheless, we wish to note that while our renewed analysis and excavations corroborate Ussishkin’s (2011) claim for the existence of more than one Iron Age phase in Area D at Tel Goren (see already Stern and Matskevich 2007: 73), the stratigraphic and artefactual evidence that we have gathered so far does not enable us to date the foundation of Stratum V prior to the mid-7th century BCE (for a detailed discussion, see Mashiach 2022).

10 The evidence for an early Judahite occupation in En-Gedi may also necessitate a reevaluation of the group of vessels from Herbert E. Clark’s collection, published by the Mazar expedition (Mazar, Dothan and Dunayevsky 1966: Figs. 29–33). These vessels, allegedly looted from tombs in the area of En-Gedi, were dismissed by Stern as unrelated to the oasis due to the presence of various Iron IIB forms that are completely absent from Tel Goren Stratum V (Stern 2007: 362; see also Maeir 2007: 126). The proposed date for the En-Gedi Spring site suggests that, at least on chronological grounds, the Clark collection could have derived from En-Gedi (see also Ussishkin 2011: 229).

## References

[CIT0001] Abel, F.M. 1911. *Une croisière autour de la Mer Morte*. Paris.

[CIT0002] Aharoni, Y. 1958. Archaeological Survey of ꜤEin Gedi. *Bulletin of the Israel Exploration Society* 22: 27–45 (Hebrew), VI–VII (English abstract).

[CIT0003] Aharoni, Y. 1964. Meṣad Gozal. *RB* 72: 562–563.

[CIT0004] Aharoni, Y. 1965. Meṣad Gozal. *IEJ* 14: 112–113.

[CIT0005] Aharoni, M. and Aharoni, Y. 1976. The Stratification of Judahite Sites in the 8th and 7th Centuries BCE. *BASOR* 224: 73–90.

[CIT0006] Amit, D. 1992. Hebron—ꜤEn Gedi, Survey of Ancient Road. *EI* 23: 345–362 (Hebrew), 158* (English abstract).

[CIT0007] Avigad, N. and Geva, H. 2000. Iron Age II Strata 9–7. In: Geva, H., ed. *Jewish Quarter Excavations in the Old City of Jerusalem, Conducted by Nahman Avigad, 1969–1982, Vol. I. Architecture and Stratigraphy: Areas A, W and X-2, Final Report*. Jerusalem: 44–82.

[CIT0008] Bar-Adon, P. 1972. The Judaean Desert and Plain of Jericho. In: Kochavi, M., ed. *Judaea, Samaria and the Golan, Archaeological Survey 1967–1968*. Jerusalem: 91–149 (Hebrew).

[CIT0009] Bar-Adon, P. 1975. An Early Hebrew Inscription in a Judean Desert Cave. *IEJ* 25: 226–232.

[CIT0010] Bar-Adon, P. 1989. *Excavation in the Judean Desert* (ꜤAtiqot 9). Jerusalem (Hebrew).

[CIT0011] Barkay, G. 1992. ‘The Prancing Horse’—An Official Seal Impression from Judah of the 8th Century B.C.E. *Tel Aviv* 19: 124–129. doi: 10.1179/tav.1992.1992.1.124

[CIT0012] Barkay, G. 1995. The King of Babylonia or a Judaean Official? *IEJ* 45: 41–47.

[CIT0013] Barkay, G., Fantalkin, A. and Tal, O. 2002. A Late Iron Age Fortress North of Jerusalem. *BASOR* 328: 49–71.

[CIT0014] Ben David-Novak, H., Morin, E. and Enzel, Y. 2004. Modern Extreme Storms and the Rainfall Thresholds for Initiating Debris Flows on the Hyperarid Western Escarpment of the Dead Sea, Israel. *Geological Society of America Bulletin* 116: 718–728. doi: 10.1130/B25403.2

[CIT0015] Billig, Y., Freud, L. and Bocher, E. 2022. A Luxurious Royal Estate from the First Temple Period in Armon ha-Natziv, Jerusalem. *Tel Aviv* 49: 8–31. doi: 10.1080/03344355.2022.2056685

[CIT0016] Blake, I. 1966. Rivage occidental de la Mer Morte. *RB* 73: 564–566.

[CIT0017] Blake, I. 1967. The Dead Sea Sites of ‘The Utter Wilderness’. *The Illustrated London News* March 4: 27–29.

[CIT0018] Blecher, M. 2012. Conservation and Restoration of Plant Diversity in Oases near the Dead Sea. *Melakh Haaretz* 6: 73–102 (Hebrew).

[CIT0019] Conder, C.R. and Kitchener, H.H. 1883. *The Survey of Western Palestine: Memoirs of the Topography, Orography, Hydrography and Archaeology, Vol. 3. Judaea*. London.

[CIT0020] Cogan, M. 2021. *Under the Yoke of Ashur: The Assyrian Century in the Land of Israel*. Jerusalem.

[CIT0021] Cross, F.M. and Milik, J.T. 1956. Explorations in the Judaean BuqêꜤah. *BASOR* 142: 5–17.

[CIT0022] Dan-Goor, S. 2022. The History of Iron Age Jerusalem: A Ceramic Approach. *Tel Aviv* 49: 67–97. doi: 10.1080/03344355.2022.2057022

[CIT0023] Danin, A. 2007. Vegetation in the Dead Sea Basin. *Melakh Haaretz* 2: 39–56 (Hebrew).

[CIT0024] Davidovich, U. 2014. *The Judean Desert during the Chalcolithic, Bronze and Iron Ages (Sixth–First Millennia BCE): Desert and Sown Relations in Light of Activity Patterns in a Defined Desert Environment* (Ph.D. dissertation, The Hebrew University of Jerusalem). Jerusalem (Hebrew with English abstract).

[CIT0025] De Groot, A. and Bernick-Greenberg, H. 2012. *Excavations at the City of David 1978−1985 Directed by Yigal Shiloh, Vol. VIIB: Area E: The Finds*. Jerusalem.

[CIT0026] De Vaux, R. 1973. *Archaeology and the Dead Sea Scrolls*. Oxford.

[CIT0027] Eitam, D. 1996. The Olive Oil Industry at Tel Miqne–Ekron during the Late Iron Age. In: Eitam, D. and Heltzer, M., eds. *Olive Oil in Antiquity: Israel and Neighbouring Countries from the Neolithic to the Early Arab Period*. Padova: 167–198.

[CIT0028] Eitan, A. 1983. Vered Yericho. *Excavations and Surveys in Israel* 2: 106–107.

[CIT0029] Faust, A. 2008. Settlement and Demography in Seventh-Century Judah and the Extent and Intensity of Sennacherib’s Campaign. *PEQ* 140: 168–194. doi: 10.1179/174313008X341528

[CIT0030] Faust, A. 2021. *The Neo-Assyrian Empire in the Southwest: Imperial Domination and Its Consequences*. Oxford.

[CIT0031] Faust, A. and Weiss, E. 2011. Between Assyria and the Mediterranean World: The Prosperity of Judah and Philistia in the Seventh Century BCE in Context. In: Wilkinson, T.C., Sherratt, S. and Bennet, J., eds. *Interweaving Worlds: Systemic Interaction in Eurasia: 7th to 1st Millennia BC*. Oxford and Oakville: 189–204.

[CIT0032] Feldman, J. 1973. ‘Tomorrow go down against them, they will come up by the ascent of Ziz’: The TekoaꜤ–En Gedi Road. In: Ilan, Z., ed. *The Judean Desert and Dead Sea*. En-Gedi: 195–202 (Hebrew).

[CIT0033] Finkelstein, I. 1992. Ḥȯrvat Qiṭmīt and the Southern Trade in the Late Iron Age II. *ZDPV* 108: 156–170.

[CIT0034] Finkelstein I. 1994. The Archaeology of the Days of Manasseh. In: Coogan, M., Exum, J.C. and Stager, L.E., eds. *Scripture and Other Artifacts: Essays on the Bible and Archaeology in Honor of Philip J. King*. Louisville: 169–187.

[CIT0035] Finkelstein, I. 2020. The Emergence and Dissemination of Writing in Judah. *Semitica et Classica* 13: 269–282. doi: 10.1484/J.SEC.5.122991

[CIT0036] Finkelstein, I., Gadot, Y. and Langgut, D. 2022. The Unique Specialised Economy of Judah under Assyrian Rule and Its Impact on the Material Culture of the Kingdom. *PEQ* 154: 261–279. doi: 10.1080/00310328.2021.1949531

[CIT0037] Finkelstein, I. and Na'aman, N. 2004. The Judahite Shephelah in the Late 8th and Early 7th Centuries BCE. *Tel Aviv* 31: 60–79. doi: 10.1179/tav.2004.2004.1.60

[CIT0038] Freud, L. 2018. *Judahite Pottery in the Transitional Phase between the Iron Age and Persian Period: Jerusalem and Its Environs* (Ph.D. dissertation, Tel Aviv University). Tel Aviv (Hebrew with English abstract).

[CIT0039] Fried, L.S. 2002. The High Places (*Bāmôt*) and the Reforms of Hezekiah and Josiah: An Archaeological Investigation. *JAOS* 122: 437–465. doi: 10.2307/3087515

[CIT0040] Gadot, Y. 2015. In the Valley of the King: Jerusalem’s Rural Hinterland in the 8th–4th Centuries BCE. *Tel Aviv* 42: 3–26. doi: 10.1179/0334435515Z.00000000043

[CIT0041] Gadot, Y., Mizrahi, S., Freud, L. and Gellman, D. 2019. What Kind of Village Is This? Buildings and Agroeconomic Activities Northwest of Jerusalem during the Iron IIB–C Period. In: Lipschits, O. and Čapek, F., eds. *The Last Century in the History of Judah* (Ancient Israel and Its Literature 37). Atlanta: 89–118.

[CIT0042] Gitin, S., 1997. The Neo-Assyrian Empire and Its Western Periphery: The Levant, with a Focus on Philistine Ekron. In: Parpola, S. and Whiting, R., eds. *Assyria 1995: Proceedings of the 10th Anniversary Symposium of the Neo-Assyrian Text Corpus Project, Helsinki, September 7–11, 1995*. Helsinki: 77–103.

[CIT0043] Gitin, S. 2015. Iron Age IIC: Judah. In: Gitin, S., ed. *The Ancient Pottery of Israel and Its Neighbors, Vol. I*. Jerusalem: 345–364.

[CIT0044] Greenberg, R. and Cinamon, G. 2006. Stamped and Incised Jar Handles from Rogem Gannim and Their Implications for the Political Economy of Jerusalem, Late 8th–Early 4th Centuries BCE. *Tel Aviv* 33: 229–243. doi: 10.1179/tav.2006.2006.2.229

[CIT0045] Hadas, G. 2001–2002. Ein Gedi Water Mills. *Strata: Bulletin of the Anglo-Israel Archaeological Society* 19–20: 71–93.

[CIT0046] Hadas, G. 2007. Lime Kiln near the En-Gedi Spring. In: Stern, E. *En-Gedi Excavations I: Final Report (1961–1965)*. Jerusalem: 429–431.

[CIT0047] Hadas, G. 2012. En-Gedi Map (147). *Archaeological Survey of Israel*. Jerusalem.

[CIT0048] Harel, M. 1967. Israelite and Roman Roads in the Judean Desert. *IEJ* 17:18–26.

[CIT0049] Herzog, Z. and Singer-Avitz, L. 2015. Iron Age IIA–B: Judah and the Negev. In: Gitin, S., ed. *The Ancient Pottery of Israel and Its Neighbors, Vol. I*. Jerusalem: 213–256.

[CIT0050] Hirschfeld, Y. 2004. Excavations at ꜤEin Feshkha, 2001. *IEJ* 54: 37–74.

[CIT0051] Hirschfeld, Y. 2006. The Nabataean Presence South of the Dead Sea: New Evidence. In: Bienkovski, P. and Galor, K., eds. *Crossing the Rift: Resources, Settlements Patterns and Interaction in the Wadi Arabah*. Oxford: 167–190.

[CIT0052] Ilan, Z. 1973. Jehoshaphat’s Battle with Ammon and Moab. *Beit Mikra* 18: 205–211 (Hebrew).

[CIT0053] Karasik, A. and Smilansky, U. 2008. 3D Scanning Technology as a Standard Archaeological Tool for Pottery Analysis: Practice and Theory. *JAS* 35: 1148–1168.

[CIT0054] Katz, H. 2008. *‘Land of Grain and Wine … A Land of Olive Oil and Honey’: The Economy of the Kingdom of Judah*. Jerusalem (Hebrew).

[CIT0055] Knauf, E.A. 2007. The Glorious Days of Manasseh. In: Grabbe, L.L., ed. *Good Kings and Bad Kings: The Kingdom of Judah in the Seventh Century BCE* (The Library of Hebrew Bible/ Old Testament Studies 393). London: 164–188.

[CIT0056] Koch, I. 2018. Introductory Framework for Assyrian–Levantine Colonial Encounters. *Semitica* 60: 367–396.

[CIT0057] Lazagabaster, I.A., Ullman, M., Porat, R., Halevi, R., Porat, N., Davidovich, U. and Marom, N. 2021. Changes in the Large Carnivore Community Structure of the Judean Desert in Connection to Holocene Human Settlement Dynamics. *Scientific Reports* 11: 3548. doi: 10.1038/s41598-021-82996-6 33574447 PMC7878878

[CIT0058] Lehmann, G. 2012. Survival and Reconstruction of Judah in the Time of Manasseh. In: Berlejung, A., ed. *Disaster and Relief Management—Katastrophen und ihre Bewältigung* (Forschungen zum Alten Testament 81). Münster: 289–309.

[CIT0059] Lipschits, O. 2000. Was There a Royal Estate in En-Gedi by the End of the Iron Age and during the Persian Period? In: Schwartz, J., Amar, Z. and Ziffer, I., eds. *Jerusalem and Eretz Israel (Arie Kindler Volume)*. Tel Aviv: 31–42 (Hebrew).

[CIT0060] Lipschits, O. 2019. The Long 7th Century BCE: Archaeological and Historical Perspectives. In: Lipschits, O. and Čapek, F. eds. *The Last Century in the History of Judah* (Ancient Israel and Its Literature 37). Atlanta: 9–43.

[CIT0061] Lipschits, O. and Čapek, F. 2019. *The Last Century in the History of Judah* (Ancient Israel and Its Literature 37). Atlanta.

[CIT0062] Lipschits, O., Sergi, O. and Koch, I. 2011. Judahite Stamped and Incised Jar Handles: A Tool for Studying the History of Late Monarchic Judah. *Tel Aviv* 38: 5–41. doi: 10.1179/033443511x12931017059468

[CIT0063] Maeir, A.M. 2007. Review of Stern, E., *En-Gedi Excavations I: Final Report (1961–1965)*. *IEJ* 57: 125–127.

[CIT0064] Magen, Y. and Peleg, Y. 2018. *Back to Qumran: Final Report (1993–2004)*. Jerusalem.

[CIT0065] Mashiach, A. 2022. *Oasis in Transition: A Reassessment of Late Iron Age En Gedi* (M.A. thesis, The Hebrew University of Jerusalem). Jerusalem (Hebrew).

[CIT0066] Master, D.M. 2009. From the BuqeꜤah to Ashkelon. In: Schloen, D., ed. *Exploring the Longue Durée: Essays in Honor of Lawrence E. Stager*. Winona Lake: 305–318.

[CIT0067] Maisler, B. 1949. A Sounding at En-Gedi. *Bulletin of the Jewish Palestine Exploration Society* 15: 25–28 (Hebrew), II (English abstract).

[CIT0068] Mazar, A. 1982. Iron Age Fortresses in the Judaean Hills. *PEQ* 114: 87–109. doi: 10.1179/peq.1982.114.2.87

[CIT0069] Mazar, A. 1990. Iron Age I and II Towers at Giloh and the Israelite Settlement. *IEJ* 40: 77–101.

[CIT0070] Mazar, A., Amit, D. and Ilan, Z. 1996. Hurvat Shilhah: An Iron Age Site in the Judean Desert. In: Seger, J.D., ed. *Retrieving the Past: Essays on Archaeological Research and Methodology in Honor of Gus W. Van Beek*. Winona Lake: 193–211.

[CIT0071] Mazar, B. 1976. En-Gedi. In: Avi-Yonah, M., ed. *The Encyclopedia of Archaeological Excavations in the Holy Land, Vol. II*. London: 370–378.

[CIT0072] Mazar, B. 1993. En-Gedi. In: Stern, E., ed. *The New Encyclopaedia of Archaeological Excavations in the Holy Land*, Vol. II. Jerusalem: 399–405.

[CIT0073] Mazar, B., Dothan, T. and Dunayevsky, I. 1966. *En-Gedi: The First and Second Seasons of Excavations 1961–1962* (ꜤAtiqot 5). Jerusalem.

[CIT0074] McCown, C.C. 1947. *Tell En-Naṣbeh I: Archaeological and Historical Results*. Berkeley and New York.

[CIT0075] Meshel, Z. and Ofer, A. 2008. A Judahite Fortress and a First-Century Building near the Top of the ꜤEn-Gedi Ascent. *IEJ* 58: 51–72.

[CIT0076] Na'aman, N. 1991. The Kingdom of Judah under Josiah. *Tel Aviv* 18: 3–71. doi: 10.1179/tav.1991.1991.1.3

[CIT0077] Naveh, J. 1958. The History of ꜤEn Gedi in Light of the Archaeological Survey (M.A. thesis, The Hebrew University of Jerusalem). Jerusalem (Hebrew).

[CIT0078] Ofer, A. 1998. The Desert Towns of Judah. *Cathedra* 90: 7–32 (Hebrew).

[CIT0079] Oron, A., Galili, E., Hadas, G. and Klein, M. 2015. Two Artificial Anchorages off the Northern Shore of the Dead Sea: A Specific Feature of an Ancient Maritime Cultural Landscape. *International Journal of Nautical Archaeology* 44: 81–94. doi: 10.1111/1095-9270.12077

[CIT0080] Parker, S.B. 2003. Graves, Caves, and Refugees: An Essay in Microhistory. *JSOT* 27: 259–288.

[CIT0081] Raz, E. 1983. *The Geology of the Judean Desert, En-Gedi Area*. Jerusalem (Hebrew).

[CIT0082] Robinson, E. 1841. *Biblical Researches in Palestine, Mount Sinai and Arabia Petraea, Vol. II*. London.

[CIT0083] Sharon, I. and Zarzecki-Peleg, A. 2006. Podium Structures with Lateral Access: Authority Ploys in Royal Architecture in the Iron Age Levant. In: Gitin, S., Wright, J.E. and Dessel, J.P., eds. *Confronting the Past: Archaeological and Historical Essays on Ancient Israel in Honor of William G. Dever*. Winona Lake: 145–167.

[CIT0084] Sapir, N., Ben-Ari, N., Freud, L. and Lipschits, O. 2022. History, Economy and Administration in Late Iron Age Judah in Light of the Excavations at Mordot Arnona, Jerusalem. *Tel Aviv* 49: 32–53. doi: 10.1080/03344355.2022.2056686

[CIT0085] Sapir-Hen, L. 2017. Pax Assyriaca and the Animal Economy in the Southern Levant: Regional and Local-Scale Imperial Contacts. In: Lipschits, O., Gadot, Y. and Adams, M.J., eds. *Rethinking Israel: Studies in the History and Archaeology of Ancient Israel in Honor of Israel Finkelstein*. Winona Lake: 341–353.

[CIT0086] de Saulcy, F. 1854. *Narrative of a Journey round the Dead Sea, and in the Bible Lands, in 1850 and 1851, Vol. I*. London.

[CIT0087] Stager, L.E. 1976. Farming in the Judean Desert during the Iron Age. *BASOR* 221: 145–158.

[CIT0088] Stern, E. 1994. The Eastern Border of the Kingdom of Judah in Its Last Days. In: Coogan, M., Exum, J.C. and Stager, L.E., eds. *Scripture and Other Artifacts: Essays on the Bible and Archaeology in Honor of Philip J. King*. Louisville: 399–409.

[CIT0089] Stern, E. 2001. *Archaeology of the Land of the Bible, Vol. II: The Assyrian, Babylonian, and Persian Periods (732–332 B.C.E)*. New Haven and London.

[CIT0090] Stern, E. 2007. *En-Gedi Excavations I: Final Report (1961–1965)*. Jerusalem.

[CIT0091] Stern, E. and Matskevich, S. 2007. Stratigraphy of the Excavated Areas. In: Stern, E., ed. *En-Gedi Excavations I: Final Report (1961–1965)*. Jerusalem: 69–75.

[CIT0092] Talis, S. 2013. Mezad Gozal. *Ḥadashot Arkheologiyot* 125. http://www.hadashot-esi.org.il/Report_Detail_Eng.aspx?id=5451 (last accessed on 8 March 2023).

[CIT0093] Thareani, Y. 2014. The Judean Desert Frontier in the Seventh Century BCE: A View from ꜤAroer. In: Tebes, J.M., ed. *Unearthing the Wilderness: Studies on the History and Archaeology of the Negev and Edom in the Iron Age* (ANES Supplement Series 45). Leuven: 227–265.

[CIT0094] Thareani-Sussely, Y. 2007. The ‘Archaeology of the Days of Manasseh’ Reconsidered in the Light of Evidence from the Beersheba Valley. *PEQ* 139: 69–77. doi: 10.1179/003103207x194091

[CIT0095] Tristram, H.B. 1865. *The Land of Israel: A Journal of Travels in Palestine, Undertaken with Special Reference to Its Physical Character*. Cambridge.

[CIT0096] Tufnell, O. 1953. *Lachish III: The Iron Age*. London.

[CIT0097] Ussishkin, D. 1980. The Ghassulian Shrine at En-Gedi. *Tel Aviv* 7: 1–44. doi: 10.1179/033443580788441071

[CIT0098] Ussishkin, D. 2011. The Dating of the *lmlk* Storage Jars and Its Implications: Rejoinder to Lipschits, Sergi and Koch. *Tel Aviv* 38: 220–240. doi: 10.1179/033443511x13099584885466

[CIT0099] Vaughn, A.G. 1999. *Theology, History, and Archaeology in the Chronicler’s Account of Hezekiah*. Atlanta.

[CIT0100] van der Veen, P.G. 2020. *Dating the Iron Age IIB Archaeological Horizon in Israel and Judah* (Ägypten und Altes Testament 98). Münster.

[CIT0101] Yezerski, I. 2007. Pottery of Stratum V. In: Stern, E., ed. *En-Gedi Excavations I: Final Report (1961–1965)*. Jerusalem: 86–129.

[CIT0102] Zimhoni, O. 1990. Two Ceramic Assemblages from Lachish Levels III and II. *Tel Aviv* 17: 3–52. doi: 10.1179/tav.1990.1990.1.3

